# Duplicated Copy Number Variant of the Maize 9-Lipoxygenase *ZmLOX5* Improves 9,10-KODA-Mediated Resistance to Fall Armyworms

**DOI:** 10.3390/genes15040401

**Published:** 2024-03-25

**Authors:** Peiguo Yuan, Pei-Cheng Huang, Timothy K. Martin, Thomas M. Chappell, Michael V. Kolomiets

**Affiliations:** Department of Plant Pathology and Microbiology, Texas A&M University, College Station, TX 77840-2132, USA; peiguo.yuan@ag.tamu.edu (P.Y.); pei-cheng.huang@ag.tamu.edu (P.-C.H.); tim.martin@ag.tamu.edu (T.K.M.); thomas.chappell@ag.tamu.edu (T.M.C.)

**Keywords:** α-ketol, 9-hydroxy-10-oxo-12(Z),15(Z)-octadecadienoic acid (9,10-KODA), copy number variations (CNVs), 12-oxo-phytodienoic acid (12-OPDA), abscisic acid (ABA), drought tolerance, herbivory defense, JA catabolism, oxylipins

## Abstract

Extensive genome structure variations, such as copy number variations (CNVs) and presence/absence variations, are the basis for the remarkable genetic diversity of maize; however, the effect of CNVs on maize herbivory defense remains largely underexplored. Here, we report that the naturally occurring duplication of the maize 9-lipoxygenase gene *ZmLOX5* leads to increased resistance of maize to herbivory by fall armyworms (FAWs). Previously, we showed that ZmLOX5-derived oxylipins are required for defense against chewing insect herbivores and identified several inbred lines, including Yu796, that contained duplicated CNVs of *ZmLOX5*, referred to as *Yu796-2×LOX5*. To test whether introgression of the *Yu796-2×LOX5* locus into a herbivore-susceptible B73 background that contains a single *ZmLOX5* gene is a feasible approach to increase resistance, we generated a series of near-isogenic lines that contained either two, one, or zero copies of the *Yu796-2×LOX5* locus in the B73 background via six backcrosses (BC6). Droplet digital PCR (ddPCR) confirmed the successful introgression of the *Yu796-2×LOX5* locus in B73. The resulting B73-2×*LOX5* inbred line displayed increased resistance against FAW, associated with increased expression of *ZmLOX5*, increased wound-induced production of its primary oxylipin product, the α-ketol, 9-hydroxy-10-oxo-12(Z),15(Z)-octadecadienoic acid (9,10-KODA), and the downstream defense hormones regulated by this molecule, 12-oxo-phytodienoic acid (12-OPDA) and abscisic acid (ABA). Surprisingly, wound-induced JA-Ile production was not increased in B73-2×*LOX5*, resulting from the increased JA catabolism. Furthermore, B73-2×*LOX5* displayed reduced water loss in response to drought stress, likely due to increased ABA and 12-OPDA content. Taken together, this study revealed that the duplicated CNV of *ZmLOX5* quantitively contributes to maize antiherbivore defense and presents proof-of-concept evidence that the introgression of naturally occurring duplicated CNVs of a defensive gene into productive but susceptible crop varieties is a feasible breeding approach for enhancing plant resistance to herbivory and tolerance to abiotic stress.

## 1. Introduction

Maize is a major cereal crop that serves as a significant source of food, feed, and industrial products around the world. Maize yield is significantly affected by insect herbivores, including fall armyworm (FAW) [*Spodoptera frugiperda* (J.E. Smith) (Insecta: Lepidoptera: Noctuidae)], corn rootworms, aphids, and earworms [1,2,3,4]. Among these insects, FAWs are an especially highly destructive pest of not only maize but other crops like sorghum, rice, and other various grasses, resulting in enormous economic losses worldwide [5,6]. Maize is one of the most widely grown staple food crops to many African communities, covering 37 million hectares in sub-Saharan Africa [7,8]. More than 300 million people in Africa are dependent on maize for food security [9,10]. The FAW is native to the tropical and subtropical Americas, but first suddenly arrived in Africa in 2016 and spread rapidly through the continent [11,12]. FAW remains an important pest in Africa’s farming systems, leading to maize yield losses up to 58% [13,14] and estimated annual economic losses amounting to $9.4 billion in Africa alone [13]. Therefore, there is an urgent need to develop maize germplasm that can resist damage from pests, especially for the countries that do not rely on transgenic crops containing *Bacillus thuringiensis (Bt)*-genes [15,16].

The success of crop breeding programs aimed at developing varieties with superior resistance to insect herbivory is largely dependent on the identification of novel defense-related alleles, introgression of which into elite germplasm does not result in penalty for crop productivity. While it is well documented that plant resistance to chewing insects requires activation of jasmonic acid (JA) signaling pathways [17,18,19], breeding for increased production of JA does not represent a feasible strategy, as increased levels of this defense hormone often result in growth inhibition [20,21]. Recently, we have identified a novel signaling pathway in maize that governs resistance to insect herbivory mediated by an oxylipin α-ketol, named 9,10-KODA [9-hydroxy-10-oxo-12(Z),15(Z)-octadecadienoic acid], which promotes plant herbivory resistance through defense priming and direct toxicity to FAW [22,23,24]. This novel signaling molecule is the major product of a maize tonoplast-localized 9-lipoxygenase gene, *ZmLOX5* [22,24]. Mechanical wounding and insect feeding induce *ZmLOX5* expression and production of 9,10-KODA in a JA-dependent manner [22,25,26]. The transposon-insertional disruption of *ZmLOX5* markedly reduced resistance to FAW and was accompanied by reduced levels of wound-induced 12-oxo-phytodienoic acid (12-OPDA), a JA-precursor and a hormone in its own right, JA, abscisic acid (ABA), green leaf volatiles (GLVs), and insecticidal benzoxazinoids [22]. Exogenous treatment of maize seedlings with 9,10-KODA specifically induced production of 12-OPDA and ABA but not jasmonoyl-L-isoleucine (JA-Ile) and resulted in increased resistance to FAW [22]. Hence, *ZmLOX5* represents a valid molecular breeding target for the development of maize lines that are more resistant to herbivory by chewing insects.

In addition to the identification of herbivory defense-related genes, another molecular breeding strategy is to exploit genome structure variations to identify superior loci of those genes. Genome structure variation mainly includes copy number variation (CNV) and/or presence/absence variation (PAV). Maize genome is characterized by extraordinarily high frequency of genetic diversity as assayed at the level of single nucleotide polymorphisms (SNPs), insertion-deletion (InDel) polymorphisms, and CNVs. The growing evidence indicates that CNVs are among the major reasons for maize phenotypic diversity [27]. CNVs result from the genomic rearrangements that lead to gains or losses of DNA segments [28,29]. In our search for a superior *ZmLOX5* locus, we have sequenced around 1.3 Kb of the *ZmLOX5* gene spanning the last two exons in close to 400 inbred lines that represent the maize diversity panel [30]. Sequencing results indicate the presence of multiple SNPs and InDels found in the *ZmLOX5* gene. Of direct relevance to this study, Southern blotting analyses of 56 selected inbred lines identified several lines (including sweet, tropical, and temperate lines) that harbor a duplicated copy variant of *ZmLOX5*. This includes inbred Yu796, which we utilized in this study as a source of the duplicated locus, designated here as *Yu796-2×LOX5* [30].

In this study, we determined that *Yu796-2×LOX5* segregates as a single locus, indicating that *ZmLOX5* CNVs are tandemly duplicated. Next, we genetically introgressed the *Yu796-2×LOX5* locus into the B73 background via six backcrosses (BC6). The resulting *B73-2×LOX5* lines were near-isogenic to B73 and contained either a single or duplicated *ZmLOX5* locus, and they were used to rigorously test whether an increased copy number of this gene confers increased resistance to FAW. We found that maize resistance against FAW feeding was positively correlated with the gene dosage. We further showed that the duplication of *ZmLOX5* resulted in increased wound-induced expression of *ZmLOX5* and other defense-associated genes, including *ZmLOX8*, *ZmLOX10*, and *ZmJAR1a*, as well as increased wound-induced production of 9,10-KODA, 12-OPDA, and ABA.

## 2. Materials and Methods

### 2.1. Plant Materials

To replace the single *ZmLOX5* gene in B73 with the duplicated *2×LOX5* from the inbred line Yu796 [Ames27196 (Lot No.: 04ncai01 SD)], the Yu796-*2×LOX5* locus was backcrossed into the genetic background of *Mutator (Mu)* transposon-insertional *lox5-3* mutant in the B73 background at the BC7 genetic stage (B73-*lox5-3*) [22] and advanced to the BC6F3 stage to generate near-isogenic lines that segregate for duplicated *Yu796*-*2×LOX5* and the mutant locus. The backcrosses were carried out with the B73-*lox5-3* inbred rather than with B73 itself because of the ease with which the mutant locus is routinely genotyped by PCR. Homozygous duplicated CNV (*Yu796-2×LOX5/Yu796-2×LOX5*), heterozygous (*Yu796-2×LOX5*/*B73-lox5-3)* seedlings, and mutant (*B73-lox5-3/B73-lox5-3)* seedlings were identified from BCnF2 segregating populations by using PCR genotyping with *Mu*-terminal inverted repeat-specific (9242: AGAGAAGCCAACGCCAWCGCCTCYA) or gene-specific (lox5-3F: TGCCGGACCAGTCAAGCCCATAT and lox5-3R: GGCCCCTTCCGGTTCTTCAAGTC) primers, as described in the previous study [22]. Maize seeds were germinated in conical tubes (4 diameter × 20.5 height, cm) filled with commercial potting soil (Jolly Gardener Pro Line C/20 potting mix, Jolly Gardener, Atlanta, GA, USA) and grown on light shelves at room temperature (22 ± 2 °C) under 16 h light and 8 h dark light cycle.

### 2.2. Genomic DNA Extraction and Droplet Digital PCR Analyses

Individual V2-stage seedlings of B73, Yu796, and three independent B73-*2×LOX5* lines at the BC6F3 genetic stage were used for genomic DNA extraction using the ZR plant/seed DNA Kit (Zymo Research, Orange, CA, USA). Genomic DNA concentration was determined using a SpectraMax QuickDrop UV-Vis Spectrophotometer (Molecular Devices, San Jose, CA, USA). One μg genomic DNA was digested using the BamHI-HF (High-Fidelity, New England Biolabs, Ipswich, MA, USA) at 37 °C for one hour. The relative copy number of *ZmLOX5* was quantified via droplet digital PCR (ddPCR) [31] using a QX200 Droplet Digital PCR system (Bio-Rad, Hercules, CA, USA). Twenty-two µL of the samples were generated by mixing 2 µL of 20 ng/µL DNA template (digested genomic DNA) and primers with Bio-Rad ddPCR EvaGreen Supermix. Forty µL of oil emulsions of the DNA samples were generated by the Bio-Rad AutoDG automatic droplet generator (Bio-Rad, Hercules, CA, USA) and transferred to 96-well PCR plates. Plates were sealed and transferred to a Bio-Rad C1000 Touch thermocycler (Bio-Rad, Hercules, CA, USA) for PCR, using the manufacturer’s recommended protocol: 95 °C for 5 min for polymerase activation, followed by 40 cycles at 95 °C for 30 s and 60 °C for 1 minute, then 4 °C for 5 min followed by 90 °C for 5 min to stabilize the signal. Ramp time was set to 2 °C per second for each step. The 96-well plates were then moved to the QX200 droplet reader (Bio-Rad, Hercules, CA, USA) for digital classification of droplets based on fluorescence. Analysis was conducted using QX Manager Software 2.0 Standard Edition (Bio-Rad, Hercules, CA, USA). Relative copy number for *ZmLOX5* was calculated as the concentration *of ZmLOX5* DNA template divided by the concentration of the α-tubulin template [32,33]. Primer sequences are shown in Table S1.

### 2.3. Sequence Analysis of 2×LOX5 CNVs

Genomic DNA was extracted from Yu796 as described above. PCR reactions were carried out using Phusion™ High-Fidelity DNA Polymerase (Thermo Fisher Scientific™, Houston, TX, USA) and *ZmLOX5*-specific forward and reverse primers (listed in Table S1). The PCR product was loaded and separated in 1.5 % (*w*/*v*) agarose gel and purified using a gel extraction kit (QIAGEN, Germantown, MA, USA). “A-Tailing” was added to 3′ blunt-ends of 100 ng of purified PCR product using GoTaq™ DNA Polymerase (Promega, Madison, WI, USA) for 10 min at 72 °C. The gel-purified DNA fragments were cloned into a TA-vector using the TOPO™ TA Cloning™ Kit (Thermo Fisher Scientific™, Houston, TX, USA), and subsequently, the resulting plasmid constructs were transformed into TOP10 competent cells (Thermo Fisher Scientific™, Houston, TX, USA). The plasmids were extracted from the TOP10 *E. coli* strains positive for *ZmLOX5* fragment insertions and purified for the next step sequencing.

### 2.4. Mechanical Wounding of Leaves

For wound-induced gene expression and metabolite analysis, the third leaves of V3- or V4-stage *B73-2×LOX5* seedlings at the BC6F3 genetic stage and B73 inbred line were treated by crushing the leaf blade using a hemostat perpendicular to the main vein but avoiding damaging of the main vein. The whole wounded leaf region was collected at designated times as described below and immediately frozen in liquid N_2_ and stored at −80 °C freezer. For all experiments, each replicate contained at least two seedlings, and four replicates were tested for expression and metabolite analyses for each designated time point.

### 2.5. RNA Extraction and Expression Analyses Using qRT-PCR

Unwounded or wounded leaves from B73-*2×LOX5* and B73 seedlings were collected at 0, 1, 2, and 4 h post wounding (hpw). Total RNA was extracted using TRIzol™ Reagent (Invitrogen, Waltham, MA, USA) and then treated with RNase-free Dnase at 37 °C for 30 min (Ambion, Corston, Bath, UK). For qRT-PCR, transcript accumulation was measured by using the SYBR Green one-step qRT-PCR ROX Mix Kit (Thermo Fisher Scientific™, Waltham, MA, USA) and StepOne Real-Time PCR System (Thermo Fisher Scientific™, Waltham, MA, USA). The qPCR primers are listed in Table S2. Expression of a constitutively expressed house-keeping gene, *α-Tubulin* (Gene ID: Zm00001eb215710), was used as an internal control.

### 2.6. Fall Armyworm Resistance Assay

The B73-*2×LOX5* seedlings at the BC6F2 stage, segregating for individuals that were homozygous for the B73-*2×LOX5* locus (*2×LOX5/2×LOX5*), heterozygous (*2×LOX5/lox5-3* locus), and mutant (homozygous for the *lox5-3/lox5-3* mutant locus), were used for FAW clip-cage and 7-day continuous feeding assays. Seedlings of the B73 inbred line were used as the single *ZmLOX5* gene-containing controls. For the clip-cage assays, the following protocol was used. Five individual seedlings of each genotype per a replicate (four replicates total) were grown until V3- or V4-stage. FAW eggs were hatched and fed with an artificial diet as described in [22]. The insect diet contained 78.2 g/L FAW diet mix (Southland, Lake Village, AR, USA), 37 g/L casein (Muscle Feast, Hebron, OH, USA), and 13 g/L agar (BD, Sparks, MD, USA). One second- or third-instar stage larvae of FAW was confined in a “clip cage” on the third leaf for 6–8 h feeding at room temperature. To determine the leaf area removed via insect feeding, the infested leaf tissues were scanned, and the consumed leaf surface area was measured using ImageJ software (ImageJ 1.36b; Wayne Rasband, NIH, Bethesda, MD, USA). Each biological replicate contained at least five seedlings, and three replicates were tested for each designated time point. For FAW continuous infestation assay, eight FAW neonates were placed into whorls of maize seedlings that were contained in individual plastic jars, and the neonates were allowed to freely roam and feed for seven days. The consumed leaf tissue and FAW larvae weight were measured at the end of the assay. Each replicate contained at least six seedlings, and three replicates were tested.

### 2.7. Quantification of Metabolites

Unwounded or wounded leaves from B73-*2×LOX5* and B73 seedlings were collected at 0, 1, 2, and 4 hpw. Selected oxylipins and ABA were measured by using AB Sciex 3200 QTRAP LC/MS/MS (Sciex, Framingham, MA, USA) as previously described [34]. Four or five biological replicates were analyzed per each individual genotype, with each replicate containing leaves from at least two seedlings.

### 2.8. Drought Stress Test

V2-stage B73*-2×LOX5* seedlings at the BC5F3 genetic stage and from the B73 inbred line were soaked in deionized water for 1 h to achieve full water content in the soil before drought stress. Fourteen days post withholding water, the seedlings were rewatered by soaking them in deionized water for 3 h. The survival rate was calculated at 3 days post rewatering. Six biological replicates were tested per each individual genotype, with each replicate containing seven seedlings. For the short-term withholding water test, V2-stage individual seedlings were removed from conical tubes and soil was carefully removed from the roots to avoid causing damage to the root. The soil-free seedlings were weighted to obtain the value of fresh weight at 0 h (FW_0_) and wrapped with cotton at the base of the stem and then placed back in the conical tubes (Figure 7B). The measurements of fresh weight were taken at each hour post drought stress and represented in Figure 7D as FW_t_. The relative water loss was calculated using the formula

ΔFW (FW_t−1_ − FW_t_)/FW_0_,

where FW_t_ is fresh weight at each designated time point, FW_t−1_ is weight at one hour before each designated time point, and FW_0_ is weight at 0 h post drought treatment. The total relative water loss rate was calculated as

(FW_0_ − FW_17_)/FW_0_,

where FW_17_ is weight at 17 h post treatment. Five biological replicates were tested per each individual genotype, with each replicate consisting of eight seedlings. For the long-term withholding water test, the maize seeds were planted in the conical tubes, each containing an equal soil amount. V2-stage B73*-2×LOX5* seedlings at the BC5F3 genetic stage, the B73 inbred line, and the *lox5-3* mutant were soaked in deionized water for more than one hour to achieve full water content in the soil before drought stress. After soaking, each pot was covered with para-film to avoid water evaporation from the soil surface (Figure 7E). Three plants were then placed into a bigger pot as one replicate, and the pot weight was measured automatically every 20 min for 6 days, with the difference in pot weight representing the amount of water lost via transpiration. Water loss through transpiration was normalized by the average water loss in *lox5-3*. Four biological replicates (each containing three individual plants) were tested per each individual genotype.

### 2.9. Statistical Analysis

Results were analyzed using Microsoft Excel. Error bars in all the figures represent standard error (SE) of the mean value. The number of biological replicates and significance thresholds are described in the figure legends. Student’s *t*-test was carried out for comparisons between the two groups, and *p* values < 0.05 were marked with asterisks for pairs of groups in the Figures. For multiple-group analysis, statistical analysis was performed via one-way ANOVA (analysis of variance) with Tukey’s HSD (honestly significant difference) post hoc test. Pairwise differences between groups were summarized using letter labels: groups sharing the same letter label were not significantly different at *α* = 0.05, and groups with non-overlapping letter labels were significantly different at *α* = 0.05.

## 3. Results

### 3.1. Introgression of Duplicated Copy Variants of ZmLOX5 from Yu796 into B73

**Figure 1:**
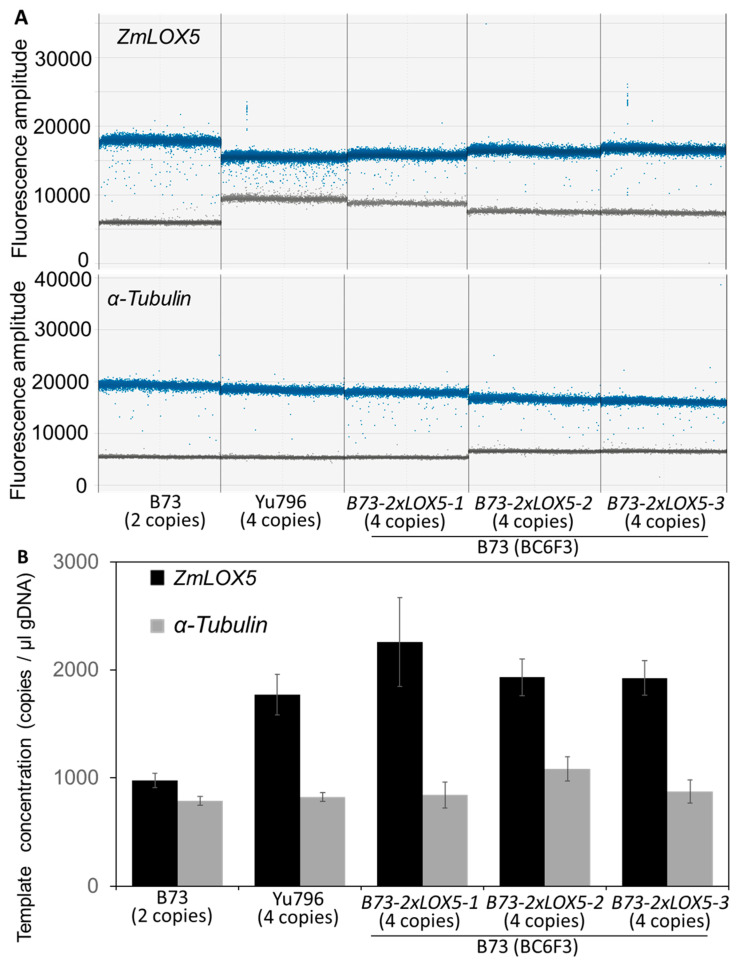

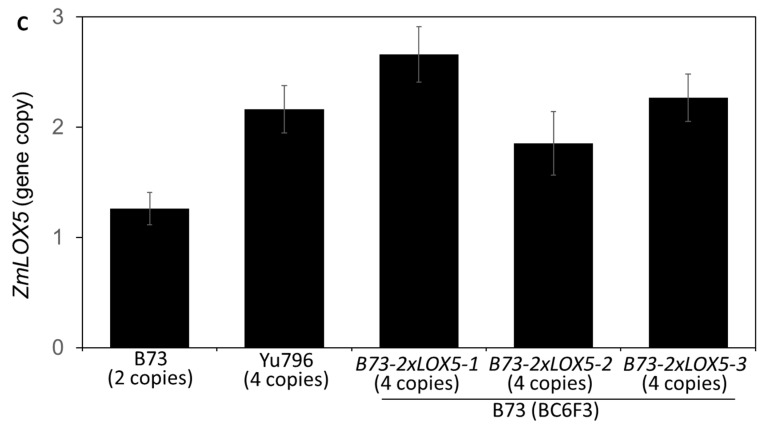
**Digital droplet PCR identification of copy number variation of *ZmLOX5* in B73 (*1×LOX5*), Yu796 (*2×LOX5*), B73-2×LOX5 genetic backgrounds of maize.** B73 inbred line carries 2 functional copies of the *ZmLOX5* gene on the two homologous chromosomes, Yu796 inbred line carries 4 functional copies of *ZmLOX5* (designated as *2×LOX5*) and three independent near-isogenic lines of B73 carrying 4 functional copies of *ZmLOX5* gene from Yu796, designated B73-*2×LOX5*, at the BC6F3 genetic stage. House-keeping single-copy gene, *α-Tubulin* (Zm00001eb215710), was chosen as an internal single copy reference. (**A**) One-dimensional plots of droplets measured for fluorescence signal (amplitude indicated on y-axis) emitted from the droplets containing either ZmLOX5 or *α-Tubulin* from each individual. Evergreen™-bound gene-positive droplets are shown in blue, while negative droplets are shown in grey. Top panel, fluorescence amplitudes of the target gene, *ZmLOX5*; bottom panel, amplitudes of the reference gene, *α-Tubulin*. (**B**) Template concentration (copies / μL) of the target gene *ZmLOX5* (black bars) and reference gene *α-Tubulin* (grey bars) in B73 and Yu796 inbred lines, as well as in three independent near-isogenic lines B73-*2×LOX5*, determined using ddPCR. (**C**) Calculated *ZmLOX5* copy numbers using the ratio of the concentrations of the target gene and reference gene (*ZmLOX5/α-Tubulin*).

To generate near-isogenic lines carrying duplicated copies of *ZmLOX5* in the B73 genetic background, we backcrossed the *Yu796-2×LOX5* locus six times into the *lox5-3* mutant, which was previously backcrossed seven times to B73 as described in [22], with selection carried out against the mutant locus at each backcross stage. This resulted in the generation of B73 lines containing the *Yu796-2×LOX5* locus, designated as *B73-2×LOX5*, that contained two CNV copies of *ZmLOX5* instead of the single *ZmLOX5* gene found in inbred B73. To confirm the gene copy number variation, we used ddPCR to test the concentration of *ZmLOX5* gene dosage. The ddPCR results show that twice as many *ZmLOX5* template molecules were found in Yu796 and the three independent near-isogenic lines of B73 carrying duplicated *ZmLOX5* at the BC6F3 genetic stage as compared to B73 (Figure 1).

Representative 1D droplet plots showed the well-defined distribution of positive (shown in blue color) and negative individual droplets (in grey color) in the upper panel for *ZmLOX5* and the lower panel for the reference gene, *α-Tubulin* (Figure 1A). As shown in Figure 1B, the copy concentration of *ZmLOX5* is 977 in B73, while almost double concentration of *ZmLOX5* is found in Yu796 (1769) and the three independent near-isogenic B73-*2×LOX5* lines (2259, 1930, and 1923, respectively). In contrast to the target gene, *ZmLOX5*, an almost equal copy number concentration was determined in each individual for the reference gene, *α-Tubulin*, representing the single copy gene. The relative copy numbers of *ZmLOX5* were calculated using *α-Tubulin* as internal controls [33,35], and the results reveal that only B73 contained a one-fold change in the *ZmLOX5*/*α-Tubulin* ratio, while Yu796 and the three independent B73-*ZmLOX5* lines contained two-fold changes in *ZmLOX5*/*α-Tubulin* ratios, indicating that these individuals carry duplicate copies of *ZmLOX5* whereas B73 encodes a single copy of *ZmLOX5*, as expected (Figure 1C).

### 3.2. CNVs of Yu796-ZmLOX5 Are Tandemly Duplicated and Contain Multiple SNPs and Several InDel When Compared to the B73-ZmLOX5 Locus

To determine whether CNVs of ZmLOX5 are tandemly duplicated (therefore, genetically linked) or interspersed (thus, not linked), we calculated the segregation ratio of *2×LOX5* in the BC4F2, BC5F2, and BC6F2 populations [29]. These F2 populations were segregated for individuals homozygous for the duplicated *ZmLOX5* locus (*2×LOX5/2×LOX5*) carrying four copies of *ZmLOX5* gene, the heterozygous locus (*2×LOX5/lox5-3*) carrying two functional copies, and the mutant locus (*lox5-3/lox5-3*) carrying zero functional copies. The genotyping results reveal that the actual ratio of *2×LOX5/2×LOX5*:*2×LOX5/lox5-3*:*lox5-3/lox5-3* individuals in the BC4F2, BC5F2, and BC6F2 populations are (1.13):(2):(0.87), (0.76):(2.17):(1.07), and (0.99):(2.16):(0.85), respectively (Table 1), which is in agreement with the fact that the *2×LOX5* CNVs are inherited as a single locus. The chi-square test showed that χ^2^ distributions for tandem duplication (segregation ratio is 1:2:1) are 0.89, 2.26, and 1.00, in the respective populations, which is less than χ2 (P_0.05,2_ = 5.99, degrees of freedom of two are associated with a *p*-value > 0.05) [36], while χ^2^ distributions for interspersed duplication (segregation ratio is 1:14:1) are 144.99, 122.41, and 117.20, respectively (Table 1). Therefore, the chi-square test agrees with the hypothesis that *2×LOX5* CNVs segregate in the normal mendelian ratio consistent with a single locus, suggesting that the two copies are linked as tandem duplicates.

**Figure 2:**
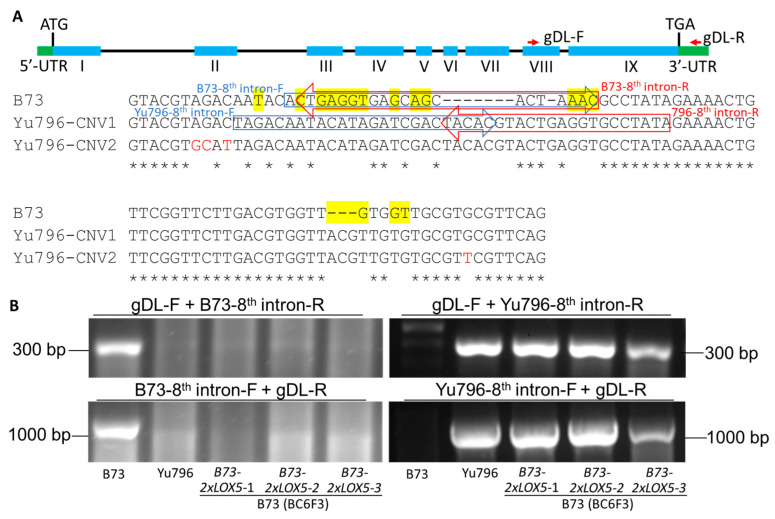
**Alignment of the nucleotide sequences of the ^8^^th^ intron of the *ZmLOX5* gene in B73 and the copy number variants (CNV) of *ZmLOX5* in Yu796 (*Yu796-CNV1* and *Yu796-CNV2*).** (**A**) Upper panel: genomic structure of the *ZmLOX5* gene containing 9 exons (shown in the blue color), 8 introns (shown in the black color), and 5′-UTR and 3′-UTR (shown in the green color). *ZmLOX5*-specific primers gDL-F and gDL-R (shown in the red color) cover partial exon VIII, complete exon IX and 3′UTR, and complete 8th intron. Sequences highlighted in the yellow color represent the nucleotide differences between the *B73-LOX5* and *Yu796-2×LOX5* locus. Nucleotides shown in the red color represent the difference between the *Yu796-CNV1* and *Yu796-CNV2* locus. Nucleotides shown in * represent the no difference between the *B73-LOX5, Yu796-CNV1* or *Yu796-CNV2*. (**B**) Genotyping of B73 and Yu796 inbred lines by using PCR analysis of genomic DNA. The row showing the B73 band represents amplicons generated by using B73 *ZmLOX5* gene-specific primers [gDL-F + B73-8th intron-R (Red arrow)] or [B73-8th intron-F (Blue arrow) + gDL-R]; the row showing the Yu-796 band represents amplicons generated by using Yu-796 *ZmLOX5* gene-specific primers [gDL-F + Yu796-8th intron-R(Red arrow)] or [Yu796-8th intron-F (Blue arrow) + gDL-R]. The line shows the genotyping background, B73, Yu796, and three independent near-isogenic lines of B73 carrying 4 functional copies of *ZmLOX5* gene from Yu796, designated B73-*2×LOX5*, at the BC6F3 genetic stage.

To characterize nucleotide sequence polymorphism between the two CNVs of *ZmLOX5*, hereafter named *ZmLOX5-CNV1* and *ZmLOX5-CNV2*, we have PCR-amplified, cloned into a TOPO TA vector, and sequenced a 1.3 Kb *ZmLOX5* fragment containing the 3′ portion of the gene spanning the partial eighth and the entire ninth exons of the *ZmLOX5* gene [30], the eighth intron, and partial 3′-UTR (the portion of the gene between the primers gDL-F and gDL-R shown in the upper panel in Figure 2A). Sequencing of the eighth and ninth exons revealed 12 SNPs differentiating *Yu796-CNV1* from *Yu796-CNV2* (Table S3). As expected, the greatest nucleotide polymorphism was identified in the eighth intron, where sequencing results reveal the presence of four SNPs differentiating *Yu796-CNV1* from *Yu796-CNV2* and sixteen SNPs that differentiate *B73-ZmLOX5* from both *Yu796-CNV1* and *Yu796-CNV2* (Figure 2A). Importantly, there were two InDels that differentiate the *B73* allele from both Yu796 CNVs that we exploited for PCR-based differentiation between the *B73-ZmLOX5* and *ZmLOX5-CNVs* by designing primers that specifically amplified either the B73 or Yu796 loci.

As shown in Figure 2B, the specific primer pairs for *ZmLOX5* in B73, (gDL-F + B73-8th intron-R) or (B73-8th intron-F + gDL-R), successfully amplified the expected PCR products using genomic DNA from B73, but not from Yu796 or three independent near-isogenic lines of B73-2×LOX5. On the contrary, the specific primer pairs for Yu796-2×*ZmLOX5*, (gDL-F + Yu796-8th intron-R) or (Yu796-8th intron-F + gDL-R) successfully amplified the PCR products using genomic DNA from Yu796 or three independent near-isogenic lines of B73-2×LOX5, but not from B73. These results demonstrate that these *ZmLOX5*-specific primers can be used to identify individual plants containing the *Yu796-2×LOX5* locus during genetic advancement of this locus into diverse germplasm via the breeding programs.

### 3.3. Duplication of ZmLOX5 Leads to the Increased Expression of ZmLOX5 and Other Wound-Inducible Oxylipin Biosynthesis Genes

**Figure 3:**
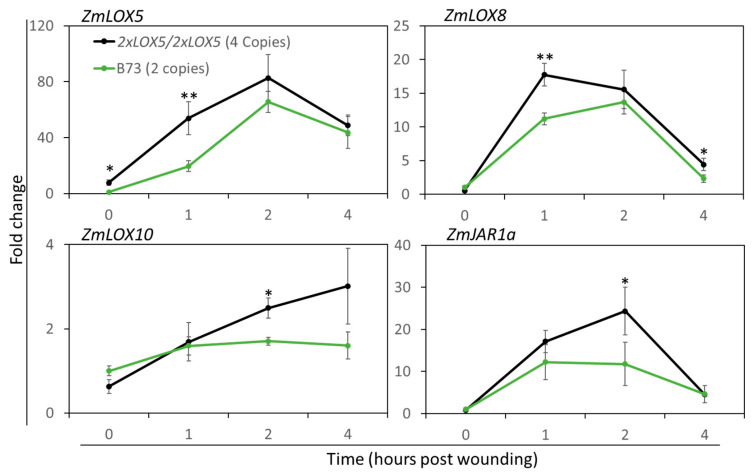
**Duplication of *ZmLOX5* in the B73 background results in enhanced expression of *ZmLOX5* and selected 12-OPDA and JA biosynthesis genes in response to wounding compared to the B73 near-isogenic line.** RT-qPCR analysis of wound-induced gene expression of *ZmLOX5*, *ZmLOX8*, *ZmLOX10*, and *ZmJAR1a* in B73 line and in the near-isogenic lines containing duplicated *ZmLOX5* CNVs in the B73 genetic background (B73-*2×LOX5*) at the BC6F3 stage at 0, 1, 2, and 4 h post wounding (hpw). The 2^−ΔΔCt^ method was used with the house-keeping gene, *α-tubulin*, utilized as internal control and compared to the mean at 0 h. For each graph, the X-axis represents hpw while the Y-axis represents expression fold change; the black line represents the B73-*2×LOX5* line (4 copies of *ZmLOX5*) and the green line represents B73 (2 copies of *ZmLOX5*); bars are means ± SE; *n* = 4. Asterisks represent significant differences between B73-*2×LOX5* and B73 lines at each time point using Student’s *t*-test (* *p* < 0.05 and ** *p* < 0.01).

It is often reported that CNVs effect the expression level of the gene by virtue of the difference in the number of functional copies [29,37]. Therefore, we measured *ZmLOX5* transcript accumulation in B73 and the *B73-2×LOX5* lines. The qRT-PCR analysis showed that *ZmLOX5* expression was increased by 7.8-fold at the resting stage and 2.8-fold at 1 hpw in the lines harboring duplicated *ZmLOX5* compared to B73 (Figure 3), indicating that the duplication of the gene resulted in enhanced expression in both untreated and wounded leaves. Unlike *ZmLOX5* at the resting stage, expression of the JA biosynthesis genes, *ZmLOX8*, *ZmLOX10*, and *ZmJAR1a* [23], are not significantly altered between *B73-2×LOX5* and B73 lines. However, in response to mechanical wounding, *ZmLOX10* and *ZmJAR1a* expression were increased 1.9- and 2.1-fold, respectively, at 2 hpw, and *ZmLOX8* expression was increased 1.6-fold at 1 hpw (Figure 3). The increased expression of *ZmLOX8*, *ZmLOX10,* and *ZmJAR1a* suggested increased biosynthesis of 12-OPDA, GLV, and/or other jasmonates.

### 3.4. Duplication of ZmLOX5 Conferred Enhanced Resistance against FAW

**Figure 4:**
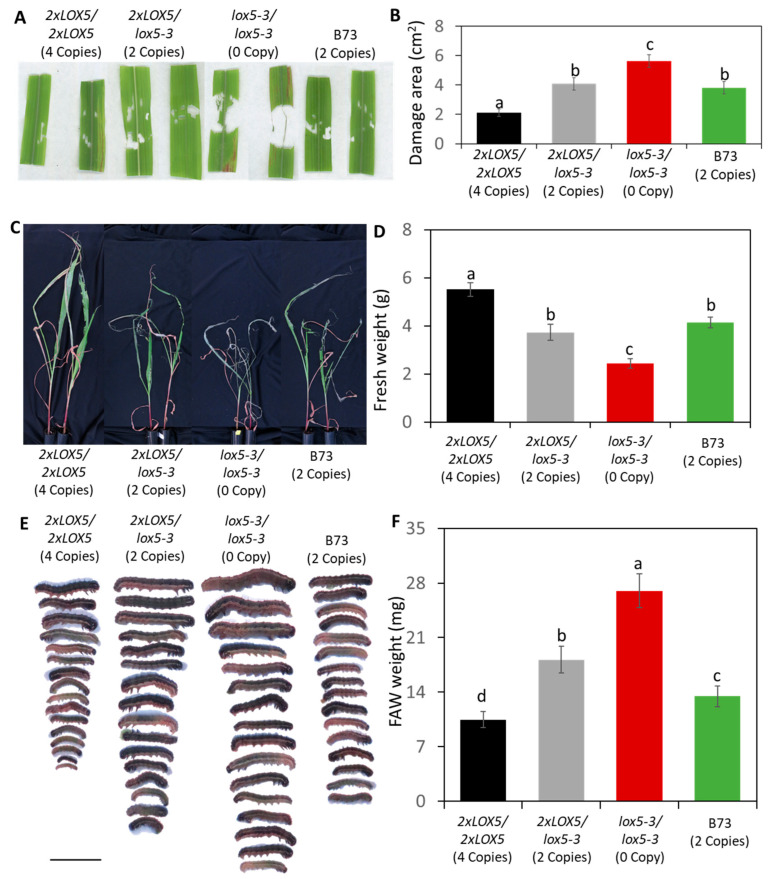
**Increased gene dosage of *ZmLOX5* results in increased resistance to fall armyworms (FAWs).** (**A**,**B**) Effect of *ZmLOX5* duplication on FAW resistance during localized herbivory in the homozygous seedlings (*2×LOX5/2×LOX5*, 4 copies), heterozygous seedlings (*2×LOX5/lox5-3*, 2 copies), knockout mutants (*lox5-3/lox5-3*, 0 copies), and B73 (2 copies). Second- to third-instar larvae were confined to clip-cages positioned on the maize leaf and allowed to feed for 6–8 h. Area consumed was determined via ImageJ analysis of scanned leaves. (**C**–**F**) The individual seedlings as in (**A**,**B**) were exposed to continued feeding by 8 neonates per seedling for 7 days. Afterwards, seedlings were photographed (**C**) and weighed (**D**). Bars are means ± SE; *n* = 6–7. The recovered larvae were photographed (**E**), and the fresh weights of recovered larvae were weighed (**F**). Bars are means ± SE; *n* = 16–20. Groups with different letters (a,b,c,d) represent statistically significant differences (*p* < 0.05) via one-way ANOVA with Tukey HSD post-hoc test.

To test whether the duplication of *ZmLOX5* increased maize resistance against insect herbivory as hypothesized, we carried out FAW resistance assays using the B73-*2×LOX5* population at the BC6F2 genetic stage, segregating for individuals containing either four copies (*2×LOX5/2×LOX5*), two copies (heterozygous, *2×LOX5/lox5-3*), or no functional copies of *ZmLOX5* (the knock-out mutant, *lox5-3/lox5-3*). The B73 inbred line with two functional copies of *ZmLOX5* (*B73-LOX5*/*B73-LOX5*) was included as a control. The individual seedlings were infested with single second- or third-instar FAW larvae placed in a clip-cage for feeding for the duration of 6–8 h. As shown in Figure 4A,B, FAW consumed the least leaf tissue in the homozygous B73-*2×LOX5* seedlings, the most leaf tissue in the mutant seedlings, and the intermediate area of leaf tissue in the heterozygous seedlings. Leaf tissue consumed by larvae in B73 was similar to the area consumed in the heterozygous seedlings (Figure 4A,B). Next, we tested FAW resistance in these same genotypes using 7-day continuous feeding assay. These assays clearly showed that the seedlings homozygous for the *2×LOX5* locus displayed the greatest resistance to FAW feeding, the mutants displayed the least resistance, and the heterozygous seedlings exhibited intermediate resistance. Similar to the assays measuring leaf area consumed, insect resistance in B73 was similar to the heterozygous (*2×LOX5*/*lox5*-3) seedlings (Figure 4C,D). These results were further supported by the measurements of FAW larvae weight gain, where we observed that larvae gained the least weight on the seedlings homozygous for the duplication, followed by the heterozygous individuals, while the most gain weight was observed on the *lox5-3* mutant, as expected (Figure 4E,F). Similarly, the larvae fed on B73 seedlings gained more weight compared to those fed on the homozygous B73-*2×LOX5* individuals (four copies) but gained less weight compared to those fed on the heterozygous individuals (two copies) (Figure 4E,F). Together, these results showed that duplication of *ZmLOX5* enhanced maize defense against FAW attack.

### 3.5. Duplication of ZmLOX5-Promoted Wound-Induced Oxylipin and ABA Production

**Figure 5:**
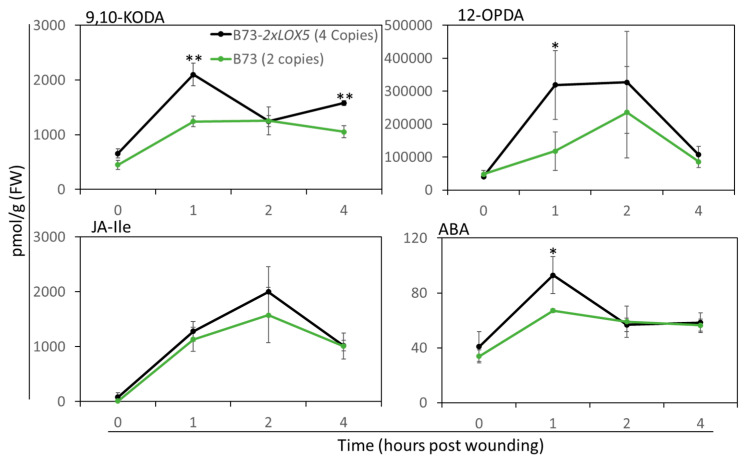
**Duplication of *ZmLOX5*-enhanced 9,10-KODA, 12-OPDA, and ABA but not JA-Ile accumulation in response to mechanical wounding.** For each graph, the X-axis represents hpw and the Y-axis represents metabolite concentration in pmol/g FW for each metabolite (JA-Ile, 12-OPDA, 9,10-KODA, and ABA) indicated within the graphs. The black line represents duplicated copy variant (*2×ZmLOX5/2×ZmLOX5*, 4 copies of *ZmLOX5* in the B73 genetic background at the BC6F3 genetic stage) and the green line represents the B73 inbred line (2 copies of *ZmLOX5*); bars are means ± SE; *n* = 4–5; asterisks represent significant differences near-isogenic *2×LOX5* and *B73-LOX5* at each time point using Student’s *t*-test (* *p* < 0.05 and ** *p* < 0.01).

Our previous study revealed that wound-triggered JA-Ile production was reduced along with 9,10-KODA, 12-OPDA, and ABA in the *lox5* mutants, resulting in decreased FAW defense [22]. Therefore, we assessed whether increased resistance to FAW in B73-*2×LOX5* seedlings resulted from increased JA accumulation. Surprisingly, wound-induced JA-Ile production in B73-*2×LOX5* was not significantly increased as compared to B73 (Figure 5), suggesting that JA-Ile is not a major reason for increased resistance. However, metabolite analyses revealed that the levels of wound-induced 9,10-KODA, the major product of ZmLOX5 [22], and ABA were significantly increased at 1 and 4 hpw, while 12-OPDA production was significantly increased at 1 hpw in the B73-*2×LOX5* seedlings compared to B73. These results are consistent with our previous study that showed that exogenous treatment with 9,10-KODA strongly induced 12-OPDA and ABA, regardless of wounding, while having a modest inhibitory effect on the wound-induced JA-Ile production [22].

**Figure 6:**
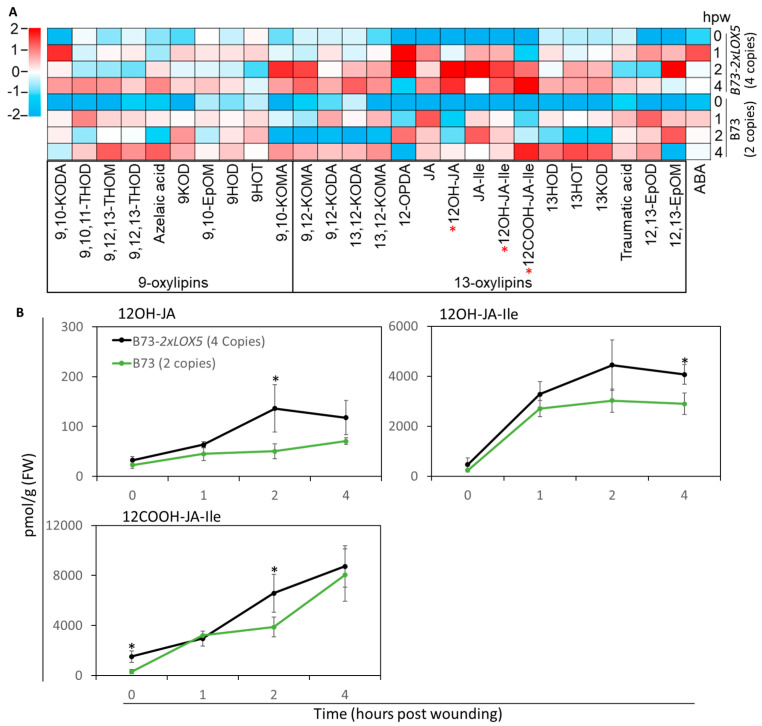
**JA catabolites 12OH-JA, 12OH-JA-Ile, and 12COOH-JA-Ile were significantly increased in the B73-*2×LOX5* seedlings in response to mechanical wounding.** (**A**) Heatmap showing relative accumulation of 9-oxylipins, 13-oxylipins, and ABA in B73-*2×LOX5*, duplicated copy variant (*2×ZmLOX5/2×ZmLOX5*, 4 copies of *ZmLOX5* in the B73 genetic background at the BC6F3 genetic stage) and B73 inbred line (2 copies of *ZmLOX5*) at 1, 2, and 4 h post wounding (hpw) compared to unwounded B73. Cells are shaded with Z-score scaling within columns, with the more abundant metabolite in red and the less abundant metabolite in blue. (**B**) For each graph, the X-axis represents hpw and the Y-axis represents metabolite concentration in pmol/g FW for each JA catabolite (12OH-JA, 12OH-JA-Ile and 12COOH-JA-Ile) indicated within the graphs. Black line represents duplicated copy variant (*2×ZmLOX5/2×ZmLOX5*, 4 copies of ZmLOX5 in the B73 genetic background at the BC6F3 genetic stage) and green line represents B73 inbred line (2 copies of *ZmLOX5*); bars are means ± SE; *n* = 4–5; asterisks represent significant differences between near-isogenic 2×LOX5 and B73-LOX5 at each time point using Student’s *t*-test (* *p* < 0.05).

Because the JA biosynthesis genes, *ZmLOX8*, *ZmLOX10*, and *ZmJAR1a*, were expressed at higher levels, but JA-Ile levels were not significantly changed as the result of *ZmLOX5* duplication, we measured the accumulation of the JA catabolites, which are not known to exhibit biological activity ascribed to JA-Ile [38,39]. Additional oxylipin profiling (Figure 6A) showed that the JA catabolites, 12OH-JA, 12OH-JA-Ile, and 12COOH-JA-Ile, were significantly increased in B73-*2×LOX5* seedlings compared to B73 (Figure 6B), suggesting that JA-Ile levels were reduced due to increased JA catabolism. In summary, these results indicate that the increased gene copy number of *ZmLOX5* enhanced FAW defense through increased production of 9,10-KODA, 12-OPDA, and ABA, but not JA-Ile.

### 3.6. Duplication of ZmLOX5-Promoted Drought Tolerance

**Figure 7:**
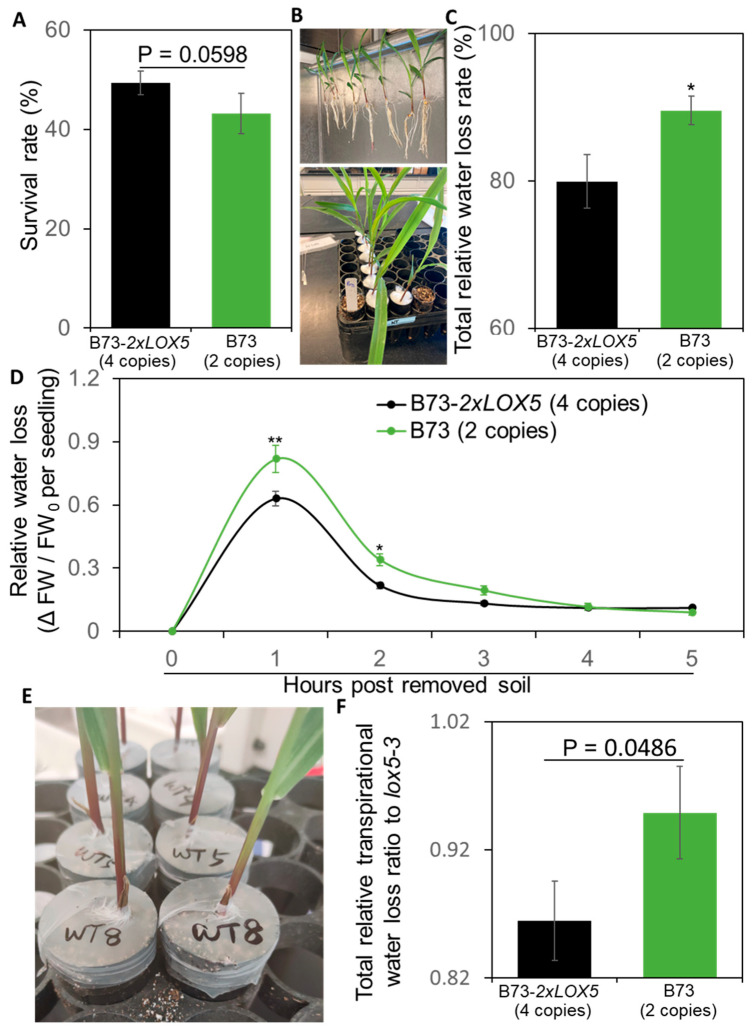

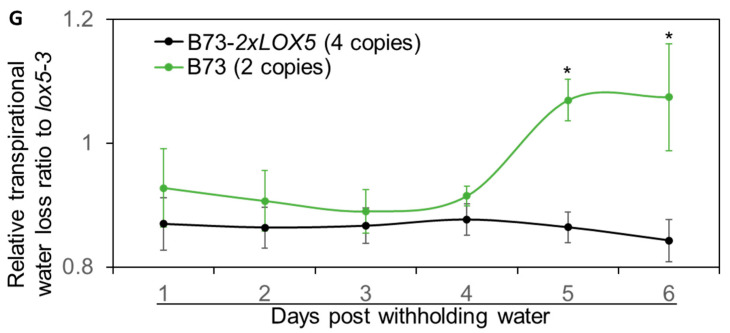
**Increased gene dosage of *ZmLOX5* results in reduced water loss during drought stress.** (**A**) Survival rates of B73-*2×LOX5* (*2×LOX5/2×LOX5*, 4 copies) and B73 inbred line after 14-day drought stress followed by recovery for 4 days of re-watering. Error bars indicate SE based on eight biological replicates. (**B**) The seedlings from B73-2×LOX5 and B73 were removed from soil and exposed to air for short-term drought stress. (**C**) The total relative water loss rates (%) of B73-*2×LOX5* and B73 inbred line at 17 h post drought treatment. (**D**) The relative water loss [ΔFW (fresh weight) (FW_t−1_ − FW_t_)/FW_0_ (fresh weight before drought stress) per maize seedling] of B73-*2×LOX5* and B73 inbred line during dehydration stress. (**E**) The seedlings from B73-2×LOX5 and B73 were wrapped with a para-film to avoid water evaporation from soil for long-term drought stress. (**F**) The total relative transpirational water loss ratio of B73-*2×LOX5* and the B73 inbred line compared to the *lox5-3* mutant at 6 days post withholding water; the relative water loss ratio of *lox5-3* was set as “1” and the ratios of B73-*2×LOX5*/*lox5-3* and B73/*lox5-3* were present. (**G**) The relative transpirational water loss of B73-*2×LOX5* and the B73 inbred line as compared to the *lox5-3* mutant during long-term drought stress; the relative water loss ratio of *lox5-3* was set as “1” and the ratios of B73-*2×LOX5*/*lox5-3* and B73/*lox5-3* were presented for each day. Asterisks represent significant differences between B73-2×LOX5 and B73 lines at each time point using Student’s *t*-test (* *p* < 0.05 and ** *p* < 0.01).

ABA and 12-OPDA play a key role in the regulation of drought tolerance in plants [40,41]. Due to the elevated stress-induced levels of ABA and 12-OPDA in B73-*2×LOX5* seedlings, we tested whether the duplication of *ZmLOX5* altered maize response to drought. The dehydration stress tests revealed that B73-*2×LOX5* seedlings exhibited moderately increased survival rate under 14 days of drought stress followed by rewatering (Figure 7A). ABA and 12-OPDA promote drought tolerance through regulation of stomatal closure [42,43]; hence, we assessed water loss in B73-*2×LOX5* and the B73 inbred line upon seedling exposure to drought (Figure 7B). As shown in Figure 7C, B73-*2×LOX5* lost less water as compared to B73 in response to seedling dehydration for 17 h. Furthermore, reduced water loss in B73-*2×LOX5* was already evident at 1 and 2 h post water deprivation (Figure 7D). To further measure whether the duplication of *ZmLOX5* enhanced relative drought tolerance levels, we determined transpirational water loss in response to long-term withholding of water (Figure 7E). Similar to the short-term dehydration test, we observed that less water was lost through transpiration in B73-*2×LOX5* compared to B73 after 6 days of withholding water (Figure 7F). These observations suggest that the duplication of *ZmLOX5* increased maize tolerance to drought stress. Further field-based tests will be needed to firmly establish whether the duplication significantly promotes drought tolerance.

## 4. Discussion

A high level of structural variation with frequent changes in the genome content was observed in diverse maize genomes. When two maize genomes were compared, more than three thousand CNV or PNV sequences were identified [27], while only several hundred CNV or PNV sequences were identified between individuals from human genomes using high-resolution study [44]. CNVs occur widely in plant genomes; however, only a few have been associated with obvious morphological, physiological, or developmental phenotypes [29,45]. One of the possible explanations is that the paralogous genes in crops usually function redundantly. As a result, the difference in copy number of one gene may lead to a change in quantitative traits, but not in the variation of qualitative traits. We observed similar results in this study. The variance of copy number of *ZmLOX5* quantitatively affected maize anti-insect resistance phenotype in a step-wise gene-dosage dependent manner. Seedlings containing four copies of *ZmLOX5* displayed the strongest insect resistance. With heterozygous *2×LOX5* (*2×LOX5* / *lox5-3*) seedlings or B73, it is important to emphasize that the B73 line harboring duplicated *ZmLOX5* CNVs was significantly more resistant to FAWs compared to the B73 inbred line, which contains a single copy of *ZmLOX5*. The increased resistance to FAW is due to increased expression of *ZmLOX5*, which in turn resulted in increased production of the major ZmLOX5 product, 9,10-KODA, that we previously showed to possess a potent signaling activity in the activation of maize defense against insect herbivores [22]. Here, we showed that the increased accumulation of 9,10-KODA was accompanied by the increased content of wound-induced levels of 12-OPDA and ABA. These results agree well with our previous study that showed that the *lox5* knock-out mutants, in addition to reduced 9,10-KODA levels, displayed reduced levels of 12-OPDA and ABA, the two defense hormones regulated by 9,10-KODA, since both of them were strongly induced by exogenous application of this α-ketol [22].

Another consistent observation is that the increased ZmLOX5-mediated antiherbivore defense does not result from increased JA production. Previously, we showed that ZmLOX5 acts downstream of JA in defense since the treatment with exogenous JA did not rescue susceptibility of *lox5* mutants [22]. Moreover, exogenous 9,10-KODA suppressed wound-induced JA-Ile production. Here, we present evidence that the lack of increased wound-induced JA production in B73-2×LOX5 can be explained by increased rate of JA catabolism. The significance of JA catabolism in the downregulation of the levels of the biologically active JA-Ile is best illustrated by the recent study of the maize *Tasselseed5* (*Ts5*) mutant. This mutant displayed a JA-deficiency phenotype, including tasselseed and reduced wound-induced JA production [38]. Positional cloning and transcriptomics analysis revealed that *ZmCYP94B1* is upregulated in *Ts5* and this gene regulated ω-oxidation of JA to convert JA or JA-Ile to 12OH-JA or 12OH-JA-Ile, respectively, resulting in the reduced JA accumulation [38]. The disruption of *12-oxo-phytodienoic acid reductases2* (*ZmOPR2*) reduced wound-induced JA and herbivory defense to FAW associated with increased JA catabolism [39]. Consistent with these studies, our data revealed that the wound-induced production of 12OH-JA, 12OH-JA-Ile, and 12COOH-JA-Ile was significantly enhanced in B73-*2×LOX5* seedlings compared to the B73 inbred line, suggesting that the *ZmLOX5*-mediated pathway promoted JA catabolism through an as of yet unknown mechanism.

Another unanticipated finding in this study is that the duplication of *ZmLOX5* may contribute to plant drought tolerance, presumably due to increased ABA and 12-OPDA production. ABA is well documented to enhance drought tolerance in plants through the regulation of stomatal closure to reduce water loss by reducing transpiration rate [42,46,47], and increasing evidence also revealed that 12-OPDA is involved in stomatal movement [43,48,49]. The potential involvement of 9,10-KODA in drought tolerance is consistent with another study that showed treatment with this molecule enhanced drought tolerance in wheat [50].

While our study provides evidence that CNVs of certain defense genes can be utilized for improving herbivory defense and drought tolerance, previous studies showed that CNVs contribute to enhancements of other plant traits. For example, increased gene copy number leads to improved resistance against soybean cyst nematodes [45]. Also, a recent review identified numerous examples of gene dosage that emerged in the course of plant adaptation to stressful environments and resulted in altered traits such as enhanced cold tolerance in grasses and enhanced herbicide resistance in weeds [51]. In maize, gene duplication effects on shaping certain traits have also been reported [52,53,54,55,56]. For example, maize plants containing a duplicated 14.6-Mb segment of chromosome 1 showed dosage-dependent effects on ear length and flowering time [53]. CNV analysis via whole genome sequencing of the lines highly resistant or highly susceptible to Goss’ bacterial wilt revealed structural genomic differences in 141 genes, including CNVs. One such CNV gene was the *rp1* rust resistance locus likely involved in resistance to this pathogen [57]. Additionally, maize plants with duplicated copies of the *tb1-ref* gene displayed increased number of crown roots and increased density of first- and second-order lateral roots [55]. Duplication at the 27-kDa γ-zein locus resulted in enhanced expression of γ-zein protein, which in turn led to endosperm modification from chalky to vitreous, yielding quality protein maize (QPM) [56]. In addition to quantitative traits, the increased gene copy number also contributes to qualitative phenotypic traits. For example, pod corn (*Tunicate* maize) is the result of dominant gain-of-function mutation at the *Tunicate (Tu)* locus. The wild-type locus contains a single-copy gene that is only expressed in leaf tissue; however, duplication of *ZMM19* in the *Tu* locus resulted in ectopic expression of the gene in the inflorescences, thereby conferring vegetative traits to the reproductive organ [52]. Overall, our study provided much needed proof-of-concept that it is possible to substantially improve maize resistance via the genetic introgression of the duplicated CNVs of *ZmLOX5* into elite germplasm that is susceptible to herbivory, such as B73.

An important aspect of this study is that we successfully adopted ddPCR technology for the accurate estimation of CNVs. This state-of-the-art method has been successfully used in determining gene copy variations of human genomic DNA [58,59] and in testing transgene copy number in several crops, including rice, citrus, potato, maize, tomato, and wheat [31,60,61]. While the original discovery of the duplicated *ZmLOX5* was based on the Southern blotting method, this method is not applicable for molecular breeding programs that rely on genotyping large numbers of individuals at each pollination event. Here, we used ddPCR to confirm the presence of duplicated *ZmLOX5* CNVs in Yu796, the original source of the *2×ZmLOX5* locus, and in B73-*2×ZmLOX5* populations. The ddPCR technology is able to accurately estimate copy number of specific DNA fragments by dividing a PCR reaction into thousands of nanoliter-scale droplets so that presence or absence of sequence of interest in a droplet is determined by the end-point fluorescence, which can be digitally counted [58]. Previously, ddPCR has been mainly used to accurately determine the number of transgenes in plants [32,60]. Because ddPCR requires the use of special instrumentation and it is rather expensive to enable its utilization in breeding programs, here, we designed primers that accurately differentiate between the *B73-LOX5* and *Yu796-LOX5* loci when used for conventional PCR. These primers may be of use in the breeding programs aimed at increasing resistance to insect herbivores to advance the duplicated *ZmLOX5* locus into the lines containing a single *ZmLOX5* gene.

## 5. Conclusions

In our previous study, we identified a novel ketol-mediated mechanism of insect herbivory resistance by the functional characterization of the *ZmLOX5* gene. In this study, we provide strong genetic evidence that naturally occurring duplication of this defense gene substantially improves resistance to the leaf-feeding insect herbivore, while simultaneously improving tolerance to drought stress. Increased gene dosage of *ZmLOX5* resulted in enhanced its transcript level and enhanced level of its product, 9,10-KODA, as well as increased herbivory-associated hormone, 12-OPDA and ABA, but not JA-Ile due to increased JA catabolism. The duplicated CNV of ZmLOX5 confer to enhanced drought tolerance through the reduction of transpirational water loss. Therefore, this study presented proof-of-concept that genetic introgression of defense-related CNVs into the productive but susceptible to herbivory and drought germplasm is a plausible breeding approach to improve these and other stress resilience traits in crops.

## Figures and Tables

**Table 1:** Duplicated copy variant of *ZmLOX5* (*2×LOX5*) segregates in the mendelian ratio consistent with a single locus, confirming that the two copies are linked as tandem duplicates, designated as copy number variant 1 (*LOX5-CNV1*) and 2 (*LOX5-CNV2*).

	*2×LOX5/* *2×LOX5* ^a^	*2×LOX5/* *lox5-3* ^a^	*lox5-3/* *lox5-3* ^a^	Actual Ratio	Chi-Square Value (χ2 ) for Tandem CNV	Fit Tandem CNV Ratio (χ2 < 5.991, DF = 2) ^b^	Chi-Square Value (χ2) for Interspersed CNV	Fit Interspersed CNV Ratio (χ2 < 5.991, DF = 2)
Tandem CNV	1	2	1	(1):(2):(1)				
Interspersed CNV	1	14	1	(1):(14):(1)				
BC4F2 ^c^	31	55	24	(1.13):(2):(0.87)	0.89	Yes	144.99	No
BC5F2 ^c^	22	63	31	(0.76):(2.17):(1.07)	2.26	Yes	122.41	No
BC6F2 ^c^	28	61	24	(0.99):(2.16):(0.85)	1.00	Yes	117.20	No

^a^ The number of plants in each category and segregation ratios are presented for the homozygous *2×LOX5* locus carrying 4 functional copies of the gene (*2×LOX5*/*2×LOX5*), heterozygous (*2×LOX5/lox5-3)* carrying 2 functional copies of the *ZmLOX5* gene, and the mutant locus (*lox5-3/lox5-3*) carrying 0 functional copies of the *ZmLOX5* gene. ^b^ χ2 (P_0.05,2_ = 5.99, degrees of freedom of 2 are associated with a *p*-value > 0.05); χ^2^ distributions for tandem duplication (segregation ratio is 1:2:1) are less than 5.99, consistent with the hypothesis that *2×LOX5* CNVs segregated in the normal mendelian ratio are consistent with a single locus, suggesting that the two copies are linked as tandem duplicates. ^c^ *2×LOX5* locus from Yu796 was backcrossed 4, 5, or 6 times into the B73 line carrying *lox5-3* mutant locus. The resulting segregating populations were PCR-genotyped and the segregation ratios were determined in BC4F2, BC5F2, and BC6F2 populations.

## Data Availability

The raw data supporting the conclusions of this article will be made available by the authors on request.

## Supplementary Materials

The following supporting information can be downloaded at https://www.mdpi.com/article/10.3390/genes15040401/s1. Table S1: Primer sequences of the genes for ddPCR, genotyping, and sequencing used in this study. Table S2: Primer sequences for qRT-PCR tests in this study. Table S3: Sequencing results of LOX5-CNV1 and LOX5-CNV2 showing several SNPs found in Exons 9 and 10 relative to the reference sequence of ZmLOX5 (Zm00001eb216870) of B73-REFERENCE-NAM-5.0 genome).
