# Supplementary material for: Duplicated Copy Number Variant of the Maize 9-Lipoxygenase ZmLOX5 Improves 9,10-KODA-Mediated Resistance to Fall Armyworms
Source: Genes (Basel). 2024 Mar 25;15(4):401. doi: 10.3390/genes15040401 (PMC11049851; doi:10.3390/genes15040401)
Supplement: Supplementary file 1 [file genes-15-00401-s001.zip › genes-2898405-supplementary.pdf]

genes MDPI

Table S1. Primer sequences of the genes for ddPCR, genotyping and sequencing used in this study

Primer name	Primer sequence (5'-3')
**ddPCR primers**	
ZmLOX5gsp-L	CTCCATATGAGCCTGGGCAGATTG
ZmLOX5gsp-R	CAAGCGTGGACTCCTCTCTC
α-Tubulin-F	GCTATCCTGTGATCTGCCCTG
α-Tubulin-R	CGCCAAACTTAATAACCCAGTA
**Genotyping primers**	
9242	AGAGAAGCCAACGCCAWCGCCTCYA
lox5-3F	TGCCGGACCAGTCAAGCCCATAT
lox5-3R	GGCCCCTTCCGGTTCTTCAAGTC
**Sequencing primers**	
ZmLOX5gDL-L	GCGGTGATCGAGCCGTTCGTAATC
ZmLOX5gDL-R	CAAGCGTGGACTCCTCTCTC
**B73 ZmLOX5 specific primers**	
B73-8th intron-F	ACTGAGGTGAGCAGCACTAAAC
B73-8th intron-R	GTTTAGTGCTGCTCACCTCAG
**Yu796 ZmLOX5 specific primers**	
Yu796-8th intron-F	TAGACAATACATAGATCGACTACAC
Yu796-8th intron-R	TATAGGCACCTCAGTACGTGTA

Table S2. Primer sequences for qRT-PCR tests in this study

Primer name	Primer (5'-3')	Tm (°C)	Product size (bp)
ZmLOX5-F	GGGCAGATTGTGTCTCGTAGTA	62	140
ZmLOX5-R	ATATTCAAGCGTGGACTCCTCT	60	140
ZmLOX8-F	CAGTACCGACAGACAGCCAT	60	163
ZmLOX8-R	GTTTCGGACCACCAAATCAA	57	163
ZmLOX10-F	ATCCTCAGCATGCATTAGTCC	59	139
ZmLOX10-R	AGTCTCAAACGTGCCTCTTGT	59	139
ZmJAR1a-F	TCTATCCGCCAAGCTGTTTC	58	150
ZmJAR1a-R	CCCCGTCATAATGCCATAGATG	61	150
Zmα-Tubulin-F	GCTATCCTGTGATCTGCCCTG	62	139
Zmα-Tubulin-R	CGCCAAACTTAATAACCCAGTA	59	139

Table S3. Sequencing results of *LOX5-CNV1* and *LOX5-CNV2* showing several SNPs found in Exons 9 and 10 relative to the reference sequence of *ZmLOX5* (Zm00001eb216870) of B73-REFERENCE-NAM-5.0 genome)

gDNA position at B73	B73	Yu796-CNV1	Percentage (%)	Yu796-CNV2	Percentage (%)	AA position at B73	B73	Yu796-CNV1	Yu796-CNV2
3810	AAG	AAG	61.5	GTG	38.5	391	K	K	V
3963	AAC	AAC	69.2	GAC	30.8	442	N	N	D
3984	GAT	GAT	69.2	TAT	30.8	449	D	D	Y
4216	ATC	ATC	38.5	ATA	61.5	496	I	I	I
4323	CCC	CCC	46.2	CGC	53.8	532	P	P	R
4326	AGG	AGG	46.2	ATG	53.8	533	R	R	M
4354	AGG	AGG	61.5	AGC	38.5	542	R	R	S
4365	ACC	ACC	69.2	AGC	30.8	546	T	T	S
4404	AAC	AAC	69.2	ATC	30.8	559	N	N	I
4410	GGG	GGG	30.8	GAG	69.2	561	G	G	E
4596	TCC	TCC	69.2	TTC	30.8	623	S	S	F

The Table presents the SNPs found in the coding sequence of Yu796 -2×LOX5 compared to the B73-LOX5 locus and the respective changes on the amino acid (AA) sequences. % represents the frequency of specific SNP occurrence in the sequenced PCR amplification products (n=13).
